# The Effect of Periodontitis on Expression of Interleukin-21: A Systematic Review

**DOI:** 10.1155/2016/3507503

**Published:** 2016-02-22

**Authors:** Archana Mootha, Sankari Malaiappan, N. D. Jayakumar, Sheeja S. Varghese, Julie Toby Thomas

**Affiliations:** Department of Periodontics, Saveetha Dental College and Hospitals, No. 162, Poonamallee High Road, Velappanchavadi, Chennai 600077, India

## Abstract

*Purpose.* Inflammation and tissue breakdown are led by an array of inflammatory destructive mediators associated with initiation and progression of inflammatory diseases like periodontitis. Current evidence shows that these inflammatory mediators have a definitive role in the pathogenesis of various systemic diseases with an inflammatory component. Interleukin-21 (IL-21) has been associated with systemic diseases like rheumatoid arthritis and Crohn's disease that follow a chronic inflammatory cascade. Similarly recent studies have associated Interleukin-21 levels with periodontitis. This systematic review was aimed to assess the levels of IL-21 in subjects with periodontitis.* Methods.* A complete literature search was done in PubMed, Medline, Science Direct, and Cochrane databases and Google Scholar based on the inclusion/exclusion criteria. Six relevant articles were procured. Full text was read individually by two reviewers and data extraction was done based on STROBE statement.* Results.* After data extraction five observational and one interventional study were obtained. All the studies showed an increased expression of IL-21 in periodontitis and the interventional study showed reduction in IL-21 levels after nonsurgical periodontal therapy (NSP).* Conclusion.* Interleukin-21 levels are higher in periodontitis than controls. With this limited evidence further longitudinal studies are required to consider this as a definitive inflammatory marker.

## 1. Introduction

Periodontitis is a chronic inflammatory condition which was initiated by gram-negative organisms present in the tooth supporting structures [1]. Disease progression occurs as a result of host-immune response to bacteria, leading to destruction of connective tissue and alveolar bone [2]. The pathogens present in the subgingival flora produce various endotoxins which are a prerequisite for periodontal disease [3]. These endotoxins in turn activate a host-immune response to the bacterial challenge by stimulating immune cells like polymorphonuclear neutrophils (PMNs), monocytes, B cells, T cells, and fibroblasts [2] to produce various inflammatory mediators such as cytokines [4, 5], acute phase proteins [6], and proteolytic enzymes [7, 8] that mediate tissue destruction. Progression of the periodontal disease can further trigger the adaptive immune mechanism for release of inflammatory mediators resulting in further periodontal breakdown.

Cytokines are important in expression of the characteristics of the immune response to bacterial endotoxins [9]. Interleukins comprise a large group of cytokines that are naturally occurring glycoproteins produced by the body [10]. They help in recruitment of neutrophils and macrophages to participate and amplify the inflammatory immune reaction [11].

The role of IL-1, IL-6, IL-8, and IL-12 has previously been established in periodontitis [12–15] proving their definitive role in periodontal destruction. Interleukin-21 (IL-21) is an inflammatory cytokine [16] mainly expressed by activated Th1 and Th17 cells which are distinct proinflammatory lineages, and not by Th2 cells in humans pointing out it to be a proinflammatory cytokine [17]. It functions via the receptor IL-21R which is a type I cytokine receptor. Recently IL-21 has gained importance as it has been associated with the pathogenesis of inflammatory breakdown in various systemic diseases like rheumatoid arthritis [18], colitis [19], and inflammatory bowel disease [20]. On account of the fact that IL-21 plays a paramount role in inflammation [21], its over production leads to amplification of local inflammation, intensifying tissue damage and destruction [22]. Evidences prove that its regulation in vivo has clinical potential in inflammatory diseases like rheumatoid arthritis [23] and is an area for active research. Since periodontitis and rheumatoid arthritis were previously associated with one another [24] this systematic review was aimed to evaluate the expression of IL-21 in periodontitis.

## 2. Materials and Methods

Based on the aim the following structured questions were formulated:

- Is there an increase in Interleukin-21 levels in periodontitis?
- Are IL-21 levels associated with severity of periodontitis?
- Is there a difference in the IL-21 levels between types of periodontitis?

### 2.1. Search Strategy

**Figure 1:**
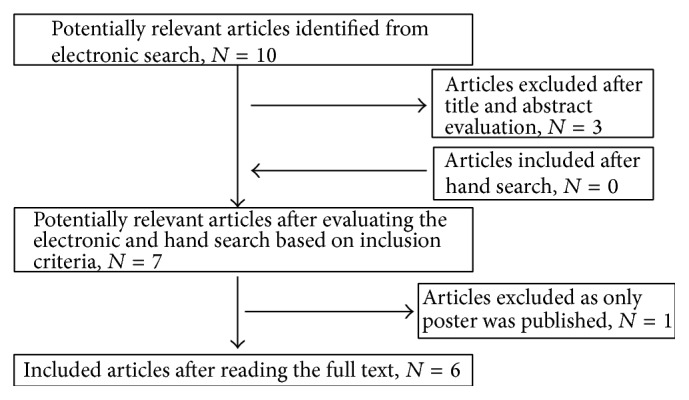
Flowchart for selection of articles.

The Cochrane, Medline/PubMed, and Science Direct databases and Google Scholar were searched to identify the relevant studies published through June 2015. A detailed search based on MeSH terms and key words was done. A hand search was carried out in the Journal of Periodontology, Journal of Periodontal Research, Journal of Clinical Periodontology, Journal of Periodontal and Implant Science, and International Journal of Periodontics and Restorative Dentistry to check for relevant additional studies. To locate additional studies, the references of the selected articles were hand-searched. No time limits and language restrictions were applied to include all the potentially relevant articles in the review. Selected studies were screened on the basis of the title and abstract. Full text was then procured for the relevant articles which fulfilled the inclusion criteria. The complete search strategy is mentioned in Table 1 and the flowchart is shown in Figure 1.

### 2.2. Inclusion and Exclusion Criteria

Studies estimating IL-21 levels in serum, saliva, gingival tissue, or GCF of subjects with chronic periodontitis or aggressive periodontitis, regardless of the methodology followed, were included. All animal studies were excluded. Studies estimating IL-21 levels in other oral lesions in subjects without periodontitis were also excluded.

### 2.3. Screening Methods and Data Extraction

The excluded and included studies are shown in Tables 2 and 3. Each study's methodological quality was assessed based on the STROBE statement by two reviewers (Archana Mootha and Sankari Malaiappan). The studies included in this review were read independently to extract the descriptive and quantitative information including citation author, year of publication, study design, study sample, number of participants in control and test groups, method of evaluation of IL-21, levels of IL-21, correlation of IL-21 levels with clinical parameters, and results.

## 3. Results

The electronic databases and hand search yielded a total of ten articles (Figure 1). Four articles [4, 25–27] were excluded and the reasons for exclusion are given in Table 2. Full texts for six articles were then procured and data extraction was done. A description of each study is given in Table 3.

The final six studies included five cross-sectional [28, 29, 31–33] and one interventional study [30]. All cross-sectional studies showed increased expression of IL-21 in periodontitis.

The first study estimated salivary IL-21 using ELISA and reported that IL-21 was expressed in all the groups (mild/moderate/no OSAS) with a significant positive correlation with CAL (*p* = 0.02) [28].

Next study [29] performed qualitative analysis of gingival tissue samples IL-21 using immunohistochemistry and quantitative analysis of salivary and gingival tissue IL-21 using western blot. Gingival tissue IL-21 (3.35 pg/mL) and GCF IL-21 (8/10) were higher in CP (chronic periodontitis) compared to controls.

Another study showed increased expression of IL-21 in gingival tissue samples using PCR, which were 1.5 times more in CP than controls (0.3) [32]. Another study [31] compared IL-21 levels in gingival tissue between CP and healthy controls using RT-PCR. CP group showed 120-fold increase of IL-21 level and showed a direct positive correlation with probing depth (*p* = 0.02) and CAL (*p* = 0.02).

One study showed reduced expression of salivary IL-21 in periodontitis using ELISA [33]. Salivary estimation of IL-21 using ELISA in CP (smokers were included) showed no significant domination of this cytokine (0.00–4.5 pg/mL) and it was concluded that the detection of IL-21 has no predictive value in health/disease.

In the interventional study, the GCF IL-21 levels were increased in CP group and there was a reduction in IL-21 levels after nonsurgical periodontal therapy when estimated using ELISA [30].

Due to variations in the study population, sample source and the methodology of estimation of IL-21 the data obtained were heterogeneous. One study was conducted in Brazilian [32], one study in Turkish [28], and one study in Chinese population [30], whereas three studies were conducted in the American [29, 31, 33] population. Varied sources were used for analysis of IL-21. Two studies used GCF [29, 30], two studies used gingival tissue [29, 31, 32], and two studies used saliva [28, 33]. All the samples showed increased IL-21 levels in periodontitis, among which GCF [30] showed maximum detection of IL-21 (80.336 pg/mL). Several methodologies were used for qualitative and quantitative analysis of IL-21. Three studies detected IL-21 using RT-PCR [31, 32], one study used western blot and immunohistochemistry [29], and three studies used ELISA [28–30, 33] for analysis of IL-21. All methodologies showed increased IL-21 levels in periodontitis, among which ELISA showed maximum detection of IL-21 [30]. Due to the heterogeneity of the data it was not feasible to pool the data to perform a meta-analysis. Only two studies showed significant correlation of IL-21 level with periodontal disease severity [31, 32]. They found a significant correlation of IL-21 with clinical attachment loss (CAL) and borderline significance between plaque index and IL-21. As CAL is the most reliable indicator of periodontal tissue destruction, this correlation indicates better that IL-21 levels increase with the severity of periodontitis.

## 4. Discussion

Periodontitis is a chronic inflammatory disease that affects the supporting structures of the teeth and is considered as one of the most common reasons for tooth loss [34]. It is one of the most common pathologies of bone and an important modifying factor of several chronic inflammatory systemic diseases like cardiovascular disease and obesity [35]. Evidence shows association of IL-21 with various systemic conditions such as coronary artery disease [36], rheumatoid arthritis [18], and inflammatory bowel disease [19]. Much about IL-21 has been studied in rheumatoid arthritis (RA), and researchers have reported elevated serum and synovial tissues levels of this cytokine in RA as the disease severity increased [37].

Periodontitis is a chronic inflammatory condition which shows increased local and systemic levels of inflammatory mediators and markers of inflammation like IL-21, and a direct link has been established between periodontitis and rheumatoid arthritis. In this regard, this systematic review aimed to evaluate the expression of IL-21 in periodontitis.

Clinically, periodontal disease is characterized by increase in probing depth and a decrease in clinical attachment level. The ultimate determinant of disease progression and clinical outcome is the immune response of host, which involves the generation of cytokines, activation of osteoclasts, and the recruitment of inflammatory cells like lymphocytes, PMNs, and antigen presenting cells to the site [2]. The recruitment and activation of these host cells lead to overproduction of proinflammatory cytokines like IL-1, IL-6, IL-12, and IL-21 along with other host-destructive mediators that enhance host destruction and activate resident cells to produce proteases which further amplify the host mediated periodontal destruction.

A limited number of eligible studies were identified in this systematic review including five observational [23, 24, 28, 29, 31–33] and one interventional studies [30]. The results of this systematic review show that five [23, 24, 28, 29, 31–33] out of six studies show an increase in IL-21 levels in chronic periodontitis subjects and one study [33] showed no increase in periodontitis. The interventional study [30] showed a reduction in IL-21 levels following nonsurgical therapy.

Nizam et al. [28] hypothesized that OSAS may predispose patients to periodontal disease and associated the salivary cytokines with OSAS severity. He found that salivary IL-6 and IL-33 were similar in OSAS groups but significantly higher than control groups, whereas IL-1*β*, IL-21, and PTX were similar in all groups with strong and weak positive correlation of IL-21 with CAL and PI, respectively. The authors concluded, it is likely that elevated IL-6 reflects the degree of subclinical inflammation in periodontal tissues, which can be a link between periodontal disease and OSAS, and the presence or severity of OSAS does not affect the level of IL-1*β* and IL-21 in either plasma or saliva.

Dutzan et al. [31] showed an increase in IL-21 levels in chronic periodontitis, a positive correlation of IL-21 with PD, CAL, IL-1*β*, IL-6, and Th17 cytokines like IL-17 and IL-23, and a negative correlation with anti-inflammatory cytokines like IL-10 and TGF-*β*1 [31].

IL-21 influences the actions of various immune cells like Natural Killer (NK) cells, PMNs, and macrophages. NK cells are a part of the innate immune response and serve as a first line of defence against invading pathogens. The development and activation of NK cells are intrinsically dependent on the activity of class I cytokine receptors, and cells treated with IL-21 displayed enhanced effector functions with greater cytolytic activity. IL-21 increased PMN migration to the local inflammatory site and caused greater PMN mediated tissue injury. IL-21 also complemented the phagocytic ability of macrophages. In addition to its action in the innate immune response, it also parades the action of the adaptive immune response. IL-21 drives terminal differentiation of B cells into plasma cells, favours the effector functions of T cell, and accelerates memory cell formation for rapid action on encounter of a pathogen for the second time. Furthermore, IL-6 and IL-21 can stimulate Th17 cells itself to produce increased IL-21. Altogether, IL-21 exaggerates the host-immune response and intensifies the local inflammatory actions, and these data support an important role of IL-21 in the pathogenesis of periodontal disease.

Salivary levels of IgA were increased in periodontitis subjects along with gingival levels of IL-21. As it was previously discussed, IL-21 not only controls Th17 activity, but also controls B cell proliferation and maturation into plasma cells; the elevated IgA levels in periodontitis may be explained, as IgA is predominantly produced by B cells and both IgA and IL-21 are increased in periodontitis compared to healthy controls. IgA is the most predominant Ig secreted by oral mucosal sites and is considered as the most important protein against microbial defence. This indicated the role of IL-21 in Ig-isotype switching in chronic periodontitis and its influence on the adaptive immune response and the immunomodulation of oral mucosa under the challenge of periodontal pathogens [32]. In contrast to these findings, Isaza et al. detected low frequency of Th17 cytokines in saliva samples and concluded that its determination is useless for the detection of disease presence and/or its severity.

After nonsurgical periodontal therapy, Zhao et al. found that Th17 cells might participate in the development of periodontitis by upregulating the expression of cytokines IL-17 and IL-21; meanwhile, Th1 cells increased the expression of IFN-*γ*, but Th2 cells inhibited the expression of IL-4. He concluded that nonsurgical treatment might decrease the expression of Th17-related cytokines, IL-17 and IL-21, and Th1-related cytokine IFN-g, while increasing the expression of Th2 related cytokine IL-4, thus contributing to the relief of periodontal inflammation [30].

While all the cross-sectional studies showed elevated IL-21 levels in periodontitis, one recent study [33] showed contradictory results by reporting no significant domination of Th17 cytokines in chronic periodontitis. An interesting finding is that there is no positive correlation of smokers with IL-21 levels. No scientific rationale explaining the results is mentioned in the article.

In addition, studies conducted in nonperiodontal oral lesions and animal studies also showed an increase in IL-21 levels in disease state compared to controls. On estimation of IL-21 levels in periapical granulomas, it was demonstrated that IL-21 along with IL-17A, TNF-*α* (tumour necrosis factor-*α*), and IFN-*γ* were higher in active periapical granulomas, while IL-4, IL-9, IL-10, and IL-22 were higher in inactive periapical granulomas. In the secondary lymphoid organs CD4+ T cell subset found in the B cell follicles was described as a major contributor to B cell-mediated antibody responses and an important source of IL-21. Since B cells are predominant in chronic lesions like periapical granulomas, it would be possible to speculate that IL-21 contributes to a Tfh-B cell response axis in periapical areas, similar to that of tertiary lymphoid tissues associated with chronic infection sites. In view of B cells being potential sources of RANKL, Tfh-B cell axis can directly drive the activity of the lesion via RANKL production [34].

Animal study conducted in rhesus monkeys by Ebersole et al. [4] reported that disease initiation/progression was characterized by overexpression of Th17/Treg cytokine genes (IL-1*β*, IL-6, TGF*β*, and IL-21) and downregulation of Th1/Th2 cytokine genes (IL-18 and IL-25). They found that several Th17/Treg cytokine genes positively correlated with tissue destruction genes (TDGs), whereas most Th1/Th2 genes exhibited a negative correlation. Overall their observations were consistent with a cytokine profile driving a Th17 type of response early in the initiation process of periodontitis, followed by a persistence with disease progression.

Isaza-Guzmán et al. [33] showed that rats fed with* Actinobacillus actinomycetemcomitans* containing mash were compared to control; both B and T cells were increased and activated with enhanced IgG switched Ig in 2 weeks. Bone resorption was evident at 12 weeks with early increase in the levels of IL-21 along with IL-2, macrophage-inhibiting factor, IL-19, TNF-*α*, and CD40 ligand, CD70, while IL-16, TNF-*α*, lymphotoxin-*β*, IFN-*α*1, and IL-1RN were increased from T cells indicating that adaptive immunity appears crucial for bone resorption.

The results of this review conclude that (1) IL-21 was elevated in subjects with chronic periodontitis compared to controls, (2) their levels were correlated with clinical severity of periodontitis (only in 2 studies, and values were given only in one study), and (3) nonsurgical periodontal therapy downregulated IL-21 levels. There are no studies based on comparison of IL-21 levels among types of periodontitis. The present review considers only published data for the interpretation of results and the raw data was not procured form the authors of unpublished studies.

This systematic review included cross-sectional studies (level III) [38] and thus indicates a low level of evidence to prove the definitive role of IL-21 in periodontitis. Future longitudinal studies are required to prove the definitive role of IL-21 in periodontitis and compare IL-21 levels among types of periodontitis. Further research in the field of Interleukin-21 could throw light on a better understanding of its role in the pathogenesis of tissue destruction in periodontitis.

## Figures and Tables

**Table 1:** Search methodology.

[[[[[[[[[[[[[[[[[[[[[[[[[[[[[aggressive periodontitis [MeSH Terms]] OR chronic periodontitis [MeSH Terms]] OR periapical periodontitis [MeSH Terms]] OR gingivitis [MeSH Terms]]] OR periodontitis] OR periodontal disease]]] AND [[[[[IL21] OR interleukin 21] OR IL-21] OR IL21 protein, human] OR Interleukin 21 protein, human]]]^*∗*^	10

^*∗*^Pubmed MESH search.

**Table 2:** Excluded studies.

S. number	Citation	Reason for exclusion
1	Ebersole et al. [4]	Animal study
2	Araujo-Pires et al. [25]	IL-21 was estimated in periapical granuloma in subjects without periodontitis
3	Li et al. [26]	Animal study
4	Wang et al. [27]	No abstract/full text available

**(a):** 

Sl. number	Author and journal	Study design	Groups	Parameters	Sample and Methodology	Statistical Analysis	Results
1	Dutzan et al. [29] Journal of Periodontology, 2011 “Levels of Interleukin-21 in Patients with Untreated Chronic Periodontitis”	Case control study	*Group 1*: controls, *n* = 19 *Group 2*: CP, *n* = 15	PI, PD, CAL, PI, BOP, and presence of IL-21	*Gingival and GCF* IL-21 (healthy-extraction site, CP- periodontal lesions) were analysed by IHC and Western blot (for presence of IL-21) and quantification was done by ELISA	Unpaired *t*-test and Mann-Whitney *U* test Chi square test	Gingival IL-21 levels were significantly higher in CP group (3.35 pg/mg) than in controls (0.98 pg/mg) (*p* < 0.05) Frequency of GCF IL-21 was greater in CP patients (8/10) than in controls (2/10) (*p* = 0.007) The Western blot and IHC staining confirmed the presence of IL-21 in periodontal tissues and GCF

**(b):** 

Sl. number	Author and journal	Study design	Groups	Parameters	Methodology	Statistical analysis	Results
2	Zhao et al. [30] Journal of Clinical Periodontology, 2011 “Effect of Non-Surgical Periodontal Therapy on the Levels of Th17/Th1/Th2 Cytokines and Their Transcription Factors in Chinese Chronic Periodontitis Patients”	Interventi-onal study	30 participants with CP, *n* = 30	IL-21	*GCF* (before and after treatment) IL-21 was analysed by ELISA	Linear correlation Paired *t*-test	Downregulation of IL-21 (80.3360 ± 39.4188 versus 31.7741 ± 13.2558 pg/mL) (*p* < 0.05) The quantity of Th17 cells in peripheral blood was decreased especially in IL-17 IFN-*γ*1 subgroup (*p* < 0.05)

**(c):** 

Sl. number	Author and journal	Study design	Groups	Parameters	Methodology	Statistical analysis	Results
3	Napimoga et al. [32] Scandinavian Journal of Immunology 2011 “Possible Involvement of IL-21 and IL-10 on Salivary IgA Levels in Chronic Periodontitis Subjects”	Case control study	*Group 1*: healthy controls, *n* = 15 *Group*2: CP, *n* = 15	IL-21	*Gingival* IL-21 (healthy-gingivoplasty sites, CP-extraction sites due to advanced periodontitis) using RT-PCR	Student's *t*-test	The mRNA levels for IL-21 was higher in the CP (1.5-fold) when compared to the healthy group (0.4-fold) (*p* < 0.05)

**(d):** 

Sl. number	Author and journal	Study design	Groups	Parameters	Methodology	Statistical analysis	Results
4	Dutzan et al. [31] Journal of Periodontology, 2012 “Interleukin-21 Expression and Its Association with Proinflammatory Cytokines in Untreated Chronic Periodontitis subjects”	Case control study	*Group 1*: controls, *n* = 8 *Group 2*: CP, *n* = 10	IL-21 mRNA level	*Gingival* IL-21 (healthy-extraction site, CP- periodontal lesions) was analysed using RT-PCR	The RQ expressed as fold change for each studied cytokine Spearman rank correlation test	A significant overexpression of IL-21 in CP affected tissues compared to healthy gingival tissues (120-fold) Significant positive correlations of IL-21 with PD (0.71) (*p* = 0.002) and CAL (0.60) (*p* = 0.01)

**(e):** 

Sl. number	Author and journal	Study design	Groups	Parameters	Methodology	Statistical analysis	Results
5	Isaza-Guzmán et al., 2015 [33] Journal of Archives of Oral Biology “Association Study between Salivary Levels of Interferon (IFN)-Gamma, Interleukin (IL)-17, IL-21, and IL-22 with Chronic Periodontitis”	Cross-sectional study	*Group 1*: CP, *n* = 105 *Group 2*: healthy controls, *n* = 44	IL-21 and IL-21R	*Salivary* IL-21 and levels were analysed using RT-PCR	Pearson's chi square test	In healthy controls IL-21 (0.00–4.54) than CP (0.00 pg/mL) (*p* = 0.470) Low detection frequency of Th17 cytokines in saliva No significant domination in periodontitis

**(f):** 

Sl. number	Author and journal	Study design	Groups	Parameters	Sample and methodology	Statistical analysis	Results
6	Nizam et al. [28] Journal of Periodontology, 2014 “Salivary Cytokines and the Association between Obstructive Sleep Apnea Syndrome and Periodontal Disease”	Case-control study	*Group 1*: controls (non- OSAS), *n* = 13 *Group 2*: mild/moderate OSAS, *n* = 17 *Group 3*: severe OSAS, *n* = 22	PI, PD, CAL, BOP, and *salivary* IL-21	Salivary IL-21 was assessed by ELISA	Spearman *r* rank correlation analysis	Significant correlation was between CAL and IL-21 (*r* = −0.347) (*p* = 0.02) Borderline significance between PI and IL-21 (*r* = −0.287) (*p* = 0.05) IL-21 levels in healthy controls, moderate OSAS, and severe OSAS (90 pg/mL, 85 pg/mL, and 75 pg/mL)

IL: interleukin, OSAS: obstructive sleep apnea syndrome, GCF: gingival crevicular fluid, GI: Gingival Index, PPD: probing pocket depth, PD: probing depth, CAL: clinical attachment level, BOP: bleeding on probing, PCR: polymerase chain reaction, RT-PCR: real time polymerase chain reaction, IHC: immunohistochemistry, and ELISA: enzyme linked immunosorbant assay.
